# Supplementary material for: Two Novel Hydrate Salts of Norfloxacin with Phenolic Acids and Their Physicochemical Properties
Source: Antibiotics (Basel). 2024 Sep 14;13(9):888. doi: 10.3390/antibiotics13090888 (PMC11429011; doi:10.3390/antibiotics13090888)
Supplement: Supplementary file 1 [file antibiotics-13-00888-s001.zip › antibiotics-3189234-supplementary.pdf]

Two Novel Hydrate Salts of Norfloxacin with Phenolic Acids, and Their Physicochemical Properties

Di Liang [1], Fei Li [1], Jingzhong Duan [1], Wei Sun [1], and Xiaoyan Yu [1,*]

[1] School of Pharmaceutical Sciences, Jilin University, Changchun 130021, China; liangdi@jlu.edu.cn (D.L.); lif23@mails.jlu.edu.cn (F.L.); duanjz22@mails.jlu.edu.cn (J.D.); wsun@jlu.edu.cn (W.S.).
* Correspondence: yuxy@jlu.edu.cn ;

Supplementary Material

Table S1. Crystal data and structure refinement of salt **1** and salt **2**.

Table S2. Hydrogen Bonding Geometry for salt **1** and salt **2** (Å).

Figure S1. (a) 2D structure of salt **1** without NORF. (b) 3D structure of salt **1**. (c) π−π (centroid-to-centroid bond distance) and C–H···π (H-to-centroid bond distance) interactions of salt **1**.

Figure S2. (a) 2D structure of salt **2** without part of NORF. (b) 3D structure of salt **2**. (c) π−π (centroid-to-centroid bond distance) and C–H···π (H-to-centroid bond distance) interactions of salt **2**.

Figure S3. FT- IR spectra of two salts (a) salt **1** and pure components

(b) salt **2** and pure components.

Table S1. Crystal data and structure refinement of salt **1** and salt **2**.

	salt **1**	salt **2**
Empirical formula	$C_{23}H_{28}FN_3O_9$	$C_{24}H_{30}FN_3O_9$
Formula weight	509.48	523.51
Crystal system	monoclinic	monoclinic
Space group	$P2_1/c$	$P2_1/c$
Temperature (K)	290 (2)	298 (2)
a/Å	19.8129(6)	7.0736(13)
b/Å	9.3112(3)	18.220(4)
c/Å	12.7998(4)	19.476(4)
α/°	90	90
β/°	100.0420(10)	94.825(14)
γ/°	90	90
V/Å^3	2325.16(13)	2501.3(9)
Z	4	4
F(000)	1072	1104
crystal size (mm)	0.20, 0.20, 0.05	0.30, 0.08, 0.05
θ range /°	2.265 to 68.519	3.327 to 68.350
tot., uniq. data, R (int)	23557, 4266, 0.051	29018, 4566, 0.132
observed data [I > 2σ(I)]	3706	3187
R indices, goodness-of-fit	R_1 = 0.041, wR_2 = 0.119, S = 1.03	R_1 = 0.059, wR_2 = 0.177, S = 1.06
ρ(max/min), e/Å^3	0.22/-0.22	0.28/-0.22
CCDC No.	2377031	2377032

Table S2. Hydrogen Bonding Geometry for salt **1** and salt **2** (Å).

D–H···A	*d*(D-H)/(Å)	*d*(H···A)/(Å)	*d*(D···A)/(Å)	<D-H···A/°	Symmetry code
			salt **1**		
O6-H6···O5	0.82	1.723	2.492	155.34	
N1-H1A···O11	0.89	1.968	2.806	156.42	[-x, -y+2, -z-1]
N1-H1B···O12	0.89	1.912	2.775	162.86	[-x, -y+3, -z-1]
C23-H23B···F4	0.97	2.27	2.886	120.47	
C35-H35C···O5	0.96	2.6	3.221	122.62	[-x-1, -y+2, -z-1]
O9-H9···O7	0.82	1.842	2.648	167.26	[x, -y+5/2, z-1/2]
O10-H10···O00D	0.82	2.485	3.261	158.2	[x+1, y+1, z]
O10-H10···O6	0.82	2.233	2.911	140.2	[x+1, y+1, z]
O11-H11A···O7	0.849	1.829	2.666	168.22	[-x, -y+2, -z-1]
O11-H11B···O8	0.85	1.876	2.706	164.99	
O12-H12A···O11	0.856	1.971	2.825	176.02	[-x, y+1/2, -z-3/2]
O12-H12B···O8	0.85	1.949	2.795	173.67	
			salt **2**		
O2-H2···O3	0.82	1.764	2.527	154.07	
O9-H9A···O1	0.85	1.938	2.747	158.33	[-x+1, -y+1, -z]
O9-H9B···O5	0.85	1.872	2.714	170.25	
O8-H8A···O4	0.851	2.014	2.814	156.23	[-x+1, -y+1, -z+1]
O8-H8B···O5	1.052	1.725	2.762	168.09	
C13-H14B···F1	0.97	2.184	2.857	125.41	
C24-H24B···F1	0.96	2.507	3.428	160.67	[x, -y+3/2, z+1/2]
C12-H12B···O9	0.96	2.568	3.394	144.27	[-x+1, y-1/2, -z+1/2]
C12-H12A···O3	0.96	2.604	3.407	141.33	[-x+2, -y+1, -z]
N3-H13D···O8	0.89	1.875	2.753	168.96	[x+1, y, z]
N3-H13C···O4	0.89	1.836	2.72	172.21	
N3-H13C···O5	0.89	2.649	3.311	131.99	
O7-H7···O9	0.946	1.891	2.729	146.29	[x, -y+3/2, z+1/2]

(a)

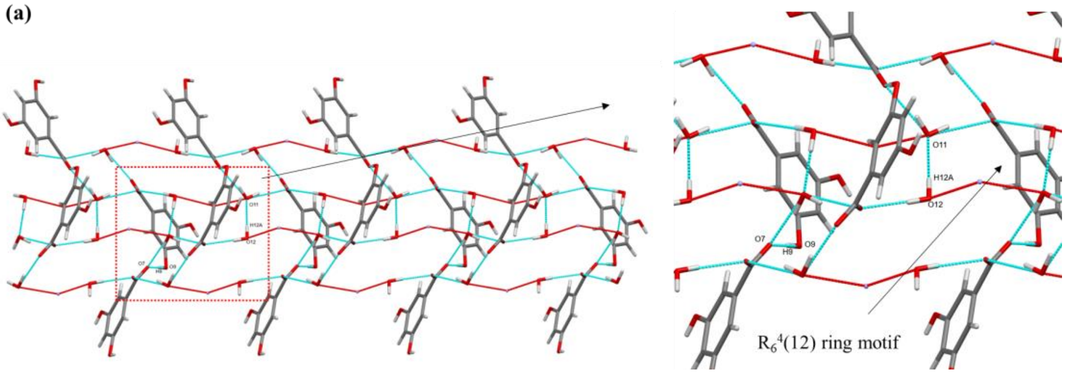

(b)

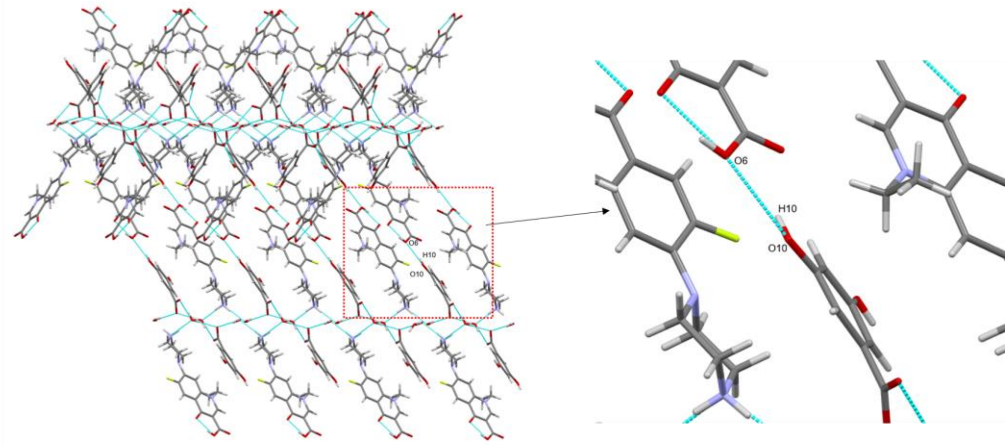

(c)

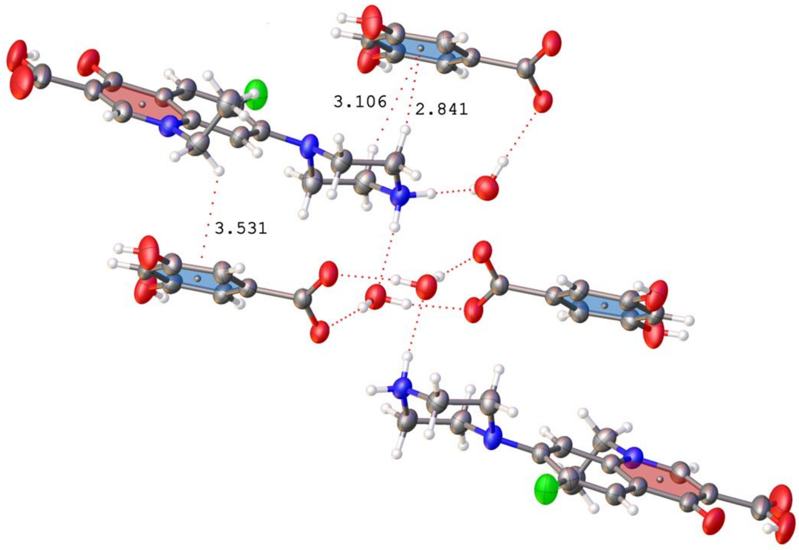

Figure S1. (a) 2D structure of salt **1** without NORF. (b) 3D structure of salt **1**. (c) C–H···π (H-to-centroid bond distance) interactions of salt **1**.

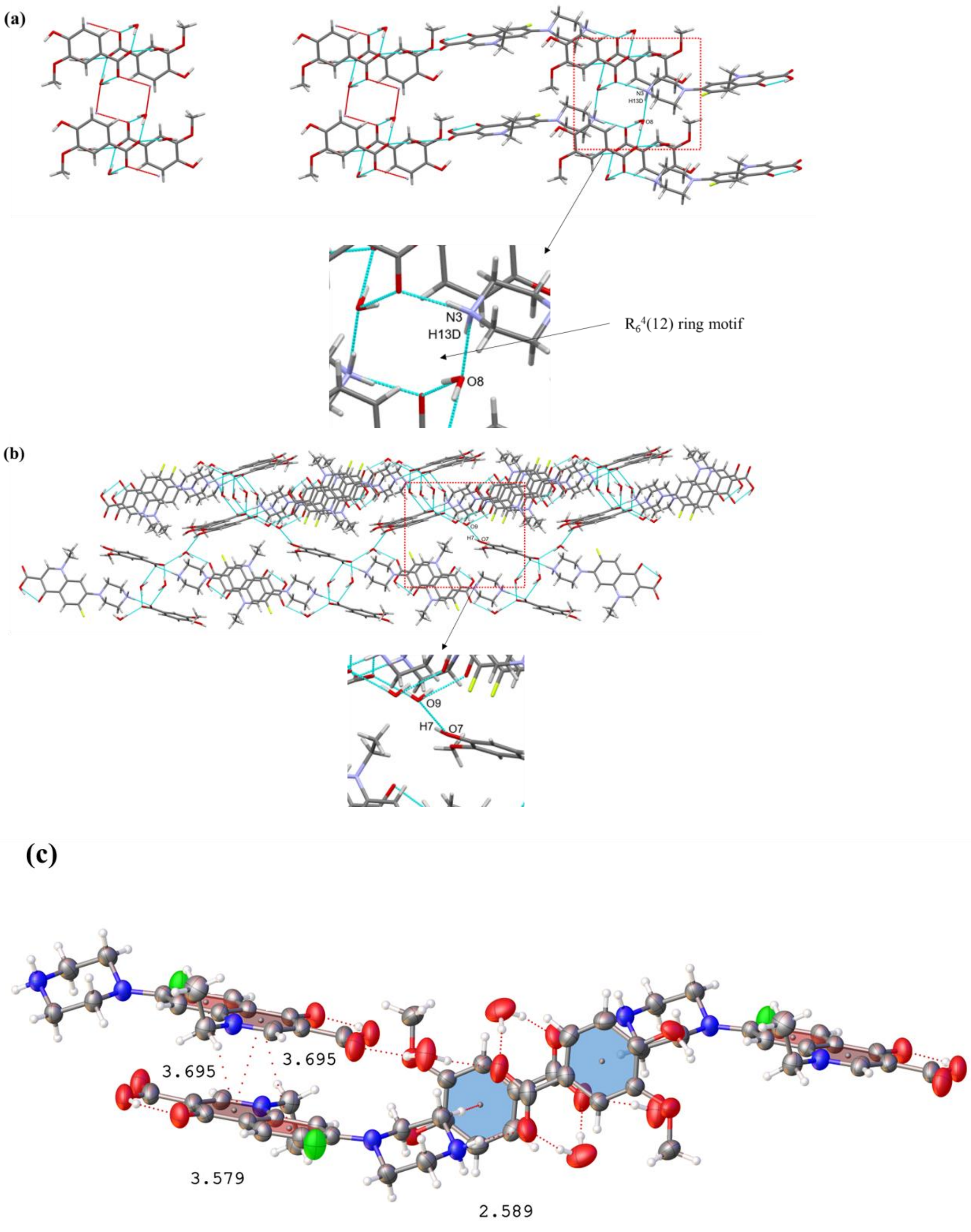

Figure S2. (a) 2D structure of salt **2** without part of NORF. (b) 3D structure of salt **2**. (c) π–π (centroid-to-centroid bond distance) and C–H···π (H-to-centroid bond distance) interactions of salt **2**.

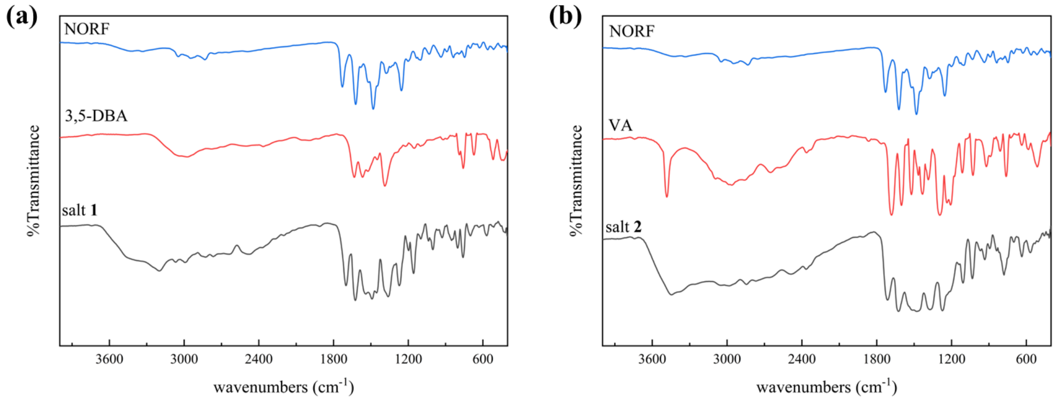

Figure S3. FT- IR spectra of two salts (a) salt **1** and pure components (b) salt **2** and pure components.
